# Comparison of MRI Sequences to Predict ATRX Status Using Radiomics-Based Machine Learning

**DOI:** 10.3390/diagnostics13132216

**Published:** 2023-06-29

**Authors:** Nabila Gala Nacul Mora, Burak Han Akkurt, Dilek Kasap, David Blömer, Walter Heindel, Manoj Mannil, Manfred Musigmann

**Affiliations:** Clinic for Radiology, University of Münster and University Hospital Münster Muenster, Albert-Schweitzer-Campus 1, 48149 Muenster, Germany; burakhan.akkurt@ukmuenster.de (B.H.A.); dilek.kasap@uni-muenster.de (D.K.); david@bloemer.cc (D.B.); walter.heindel@uni-muenster.de (W.H.); mannil@uni-muenster.de (M.M.); manfred.musigmann@dzbank.de (M.M.)

**Keywords:** neuroimaging, radiomics, artificial intelligence, MRI, glioma, ATRX

## Abstract

ATRX is an important molecular marker according to the 2021 WHO classification of adult-type diffuse glioma. We aim to predict the ATRX mutation status non-invasively using radiomics-based machine learning models on MRI and to determine which MRI sequence is best suited for this purpose. In this retrospective study, we used MRI images of patients with histologically confirmed glioma, including the sequences T1w without and with the administration of contrast agent, T2w, and the FLAIR. Radiomics features were extracted from the corresponding MRI images by hand-delineated regions of interest. Data partitioning into training data and independent test data was repeated 100 times to avoid random effects. Feature preselection and subsequent model development were performed using Lasso regression. The T2w sequence was found to be the most suitable and the FLAIR sequence the least suitable for predicting ATRX mutations using radiomics-based machine learning models. For the T2w sequence, our seven-feature model developed with Lasso regression achieved a mean AUC of 0.831, a mean accuracy of 0.746, a mean sensitivity of 0.772, and a mean specificity of 0.697. In conclusion, for the prediction of ATRX mutation using radiomics-based machine learning models, the T2w sequence is the most suitable among the commonly used MRI sequences.

## 1. Introduction

Gliomas are the most common primary tumor of the central nervous system (CNS). They originate from supporting glial cells and are a type of neuroepithelial tumor [1].

Diffuse gliomas represent around 28 percent of all brain tumors but have the highest mortality [2], which varies depending on grading, as they can be classified as either low or high grade. Nevertheless, low-grade glioma transforms into high-grade within 5–10 years of diagnosis in approximately 70 percent of cases [3].

The classification of CNS tumors is determined by the World Health Organization (WHO). Historically the subtypes and grades were based alone on histopathology. However, on the fifth and latest update of the WHO Classification published in 2021, the classification is now also based on molecular and genetic markers [4].

According to this, adult-type diffuse glioma now constitutes three categories, depending on histological and molecular characteristics [5]:

- astrocytoma (isocitrate dehydrogenase (IDH)-mutant), which can be graded as 2, 3, or 4, depending on increasing mitotic activity and the presence of necrosis, microvascular proliferation, and/or homozygous deletion of CDKN2A/CDKN2B (in grade 4). Most of these tumors show a loss of alpha thalassemia retardation syndrome X-linked gene (ATRX) [6]
- oligodendroglioma (IDH-mutant and 1p/19q-codeleted), which can be graded in 2 or 3, the latter showing high cellularity with atypia, microvascular proliferation, and increased mitotic activity. ATRX-staining is mostly retained [6]
- and glioblastoma IDH-wildtype, historically defined as high grade, with hypercellularity, microvascular proliferation, and necrosis [6]

Because of the heterogeneity of histological features, which may lead to sampling error, the more precise molecular genetic changes have taken an important role in the characterization and grading of CNS-Tumors [5], thus improving the prognostication and the treatment planning [7,8].

The previously mentioned alpha thalassemia retardation syndrome X-linked (ATRX) gene plays an important role in different tumors, including adult-type diffuse glioma. It is involved in chromatin remodeling, maintaining genomic stability [9] and represents an important molecular marker for astrocytic IDH-mutated tumors [10], correlating with improved prognosis.

For the diagnosis of glioma is magnetic resonance imaging (MRI) the diagnostic modality of choice. The standard MRI sequences for the diagnosis of brain tumors should at least include T1-weighted before and after administration of an intravenous contrast agent (T1w native and T1w CE), T2-weighted spin echo (T2w) and fluid-attenuated inversion recovery (FLAIR) [11]. Often, a stereotactic biopsy is performed for histologic diagnosis confirmation and tumor grading [12].

However, while biopsy can only analyze a portion of the tumor, MRI can assess the entire tumor volume [13]. In addition, there is a mortality after brain biopsy of 0.7 to 4 percent. The overall morbidity is 3 to 13 percent. Transient or permanent neurologic complications, seizures, and impaired consciousness are among the most common symptoms after biopsy. Cerebral hemorrhage occurs in between 0.9 and 8.6 percent of cases [14].

On the other hand, deciding on the correct therapy based on initial MRI imaging is often problematic, as a proportion of all brain-derived tumors show characteristics of low-grade glioma on MRI imaging but turn out to be high-grade glioma after biopsy [15]. The accuracy of a standardized contrast-enhanced MRI examination in differentiating, for example, between grade 2 and grade 3 astrocytoma is 30 to 50 percent [15].

Radiomics is a general term for new analysis methods that quantify image morphological criteria using data characterization algorithms in a replicable manner. This allows tumor patterns and features to be better determined. Thus, in addition to classical parameters such as volume, other properties, such as shape or heterogeneity within the tumor, can also be recorded in quantifiable parameters. This offers the chance for a non-invasive characterization of tumor suspicious lesions.

There are different radiomics features that are extracted from imaging technics, for example, MRI, with a 2-dimensional region of interest or a 3-dimensional volume of interest. These features can be subdivided into statistical (histogram-based and texture-based), model-based, transform-based, and shape-based. After being selected, features are used to build models which can predict either present variables, such as the presence or absence of a disease, or future variables, i.e., response to a certain treatment [13].

Hence, the problem is the classification of adult-type diffuse glioma using a noninvasive, but accurate method, to prevent the possible complications of a biopsy, and the aim of our study is to determine, using radiomics-based machine learning, which of the commonly used MRI sequences is best suited for predicting the biomolecular status, specifically ATRX mutation status and what accuracies can be achieved with different MRI sequences with respect to this important diagnostic question.

## 2. Materials and Methods

The study was performed in compliance with the Declaration of Helsinki and approved by the local ethics committee (Ärztekammer Westfalen-Lippe, ÄKWL 2021-596-f-S). Due to its retrospective nature, it was not possible to acquire written informed consent from the patients. As mentioned above, the ATRX status is very important for the classification of adult-type diffuse gliomas according to the latest (2021) version of the WHO classification of CNS tumors.

Basically, we are interested in predicting ATRX mutation status, i.e., distinguishing between gliomas with retained ATRX expression (ATRX not mutated) from those with loss of ATRX expression (ATRX-mutated) using radiomics-based machine learning. In this study, our main objective is to identify the most suitable MRI sequence for this purpose from commonly used MRI sequences. Therefore, we retrospectively searched our database for patients diagnosed with glioma between October 2007 and December 2021. We then adjusted the WHO Grade according to the 2021 classification.

The corresponding MRI images were generated using the following four MRI sequences: (1). T1w without the administration of a contrast agent (T1w native), (2). T1w with the administration of a contrast agent (T1w CE = contrast-enhanced), (3). T2w, and (4). Fluid attenuated inversion recovery (FLAIR).

To be able to compare the performance achievable with these sequences and thus determine the most appropriate of these sequences for predicting ATRX mutations, we included in our study cohort only patients for whom all four sequences were available. We excluded six of these 107 patients because their ATRX status was unknown. No further patients were excluded. ATRX expression was retained in 64 of these 101 remaining patients, whereas loss of ATRX expression was observed in the remaining 37 cases. The demographic characteristics of the data used to compare MRI sequences in terms of predictability of ATRX mutation status are summarized in Table 1.

### 2.1. Radiomics

**Figure 1:**
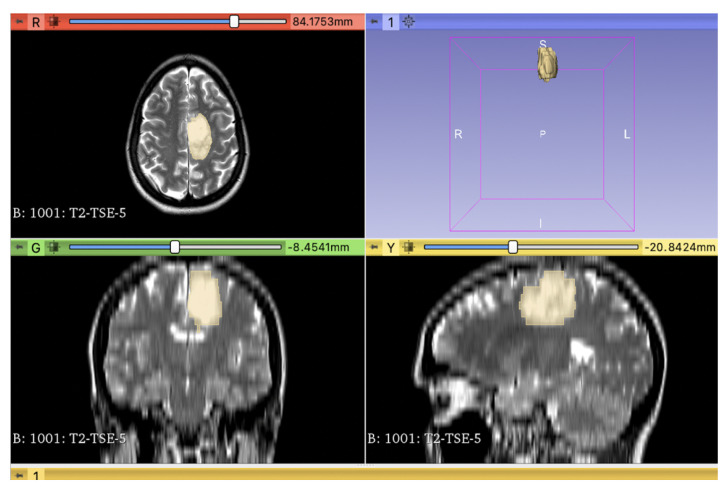
Segmentation of a T2w sequence of a Patient with IDH-mutated Oligodendroglioma frontal left, ATRX positive.

Segmentation was semi-automatically performed using the 3D Slicer open-source software platform (version 4.10, www.slicer.org, accessed on 27 February 2021) and utilizing the Segmentation Wizard plugin by two readers (see Figure 1). Expert consensus was used in anatomically difficult segmentation.

We used the open-source platform PyRadiomics, which according to van Griethuysen et al. is capable of extracting a large number of technical (radiomics) features from medical images [16]. The PyRadiomics package is available as an implementable plugin for the 3D Slicer platform. We determined a total of 107 radiomics features for each of the four MRI sequences used. The radiomics features were extracted by hand-delineated regions of interest (ROI) from the MRI images of each patient. Following the PyRadiomics documentation, the individual features can be assigned to different feature classes: (1) first order statistics (18 features), (2) shape-based (14 features), (3) gray level co-occurrence matrix (24 features), (4) gray level run length matrix (16 features), (5) gray level size zone matrix (16 features), (6) neighboring gray tone difference matrix (5 features), and (7) gray level dependence matrix (14 features). The exact calculation method for each feature can be found on the PyRadiomics website (pyradiomics.readthedocs.io, accessed on 1 October 2022). In addition to the radiomics features used, our database contained the age of the patients at the time of diagnosis and their gender as further information. All features were z-score transformed and subjected to a 95 percent correlation filter to exclude redundant information.

### 2.2. Statistical Analysis

Statistical analysis was performed using R software (version 4.1.2). R is an open-source software/programming language that is very suitable for statistical data analysis and graphical presentation of results. Special functions needed for data analysis can be easily integrated into R with the help of associated libraries/software packages. For training and testing our machine learning models, we used the R package “caret”. The area under the curve (AUC) values were calculated with the package “pROC”. All other performance values were also calculated with the caret package. We randomly assigned the 101 patients to a training group and an independent test group, using a stratified 4:1 ratio with a balanced distribution of ATRX-mutated and ATRX non-mutated patients between these two groups (see Table 1). To be able to compare the results obtained with the four different sequences used as objectively as possible, we assigned the data of all sequences (i.e., T1w native and CE, T2w and FLAIR) identically to the respective training groups or independent test groups. The training data (comprising 80 percent of the total data) were used for feature preselection and subsequent model development. Both the feature preselection and the model development were performed using least absolute shrinkage and selection operator (Lasso) regression. Lasso regression is a special form of regression analysis. By means of regularisation (so-called shrinkage), less important features lose influence or can even be completely removed from a model. The model complexity is kept as low as possible using a penalty term to avoid overfitting. Lasso regression thus independently performs a selection of suitable features/regularisation, which distinguishes this method from other regression methods such as linear regression. The methodology of Lasso regression is explained, for example, in Chintalapudi et al. [17]. Hyperparameters included in the models were optimized using 10-fold cross-validation. All models were optimized by maximizing the AUC of the receiver operator characteristic (ROC). Finally, the achieved model performance was tested using the independent test data (containing the remaining 20 percent of the total data).

We determined the discriminatory power of our models using AUC, accuracy, sensitivity, specificity, positive predictive value (PPV), and negative predictive value (NPV). Here, sensitivity describes the proportion of correctly predicted cases with retained ATRX expression and specificity describes the proportion of correctly predicted cases with loss of ATRX expression. Finally, the positive predictive value describes the proportion of correctly predicted cases with retained ATRX expression in relation to all predicted cases with retained ATRX expression. Accordingly, the negative predictive value describes the rate of correct predictions of cases with a loss of ATRX expression relative to all predictions of cases with a loss of ATRX expression.

The performance achieved by the models depends on the number of features included in the models. Therefore, to determine the optimal number of features to include in the final models and to rule out possible overfitting, we started by constructing each model multiple times with an increasing number of features (starting with a single-feature model). In each model with a predefined number of features, we included the most important features. These most important features were determined beforehand based on the training data using the “VarImp”-function in R (VarImp = variable importance). Simply described, the VarImp-function calculates the performance of a given model with and without the inclusion of a feature under investigation, and thus determines the performance gain achieved by that feature. The feature that causes the greatest performance loss when removed from the model has the highest importance. We determined the optimal number of features to include in the model by analyzing at which model complexity (in terms of the number of features included) the highest model performance was achieved with respect to the independent test data. This approach minimizes the risk of possible overfitting.

**Figure 2:**
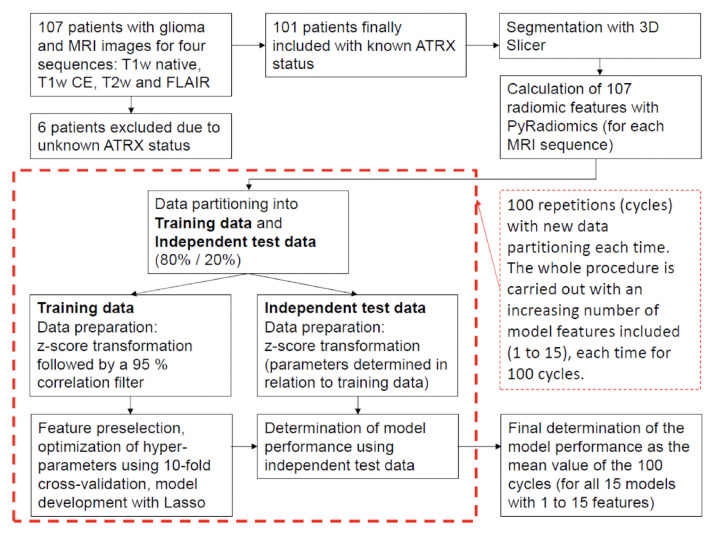
Flowchart describing the methodological approach. For each MRI sequence, a total of 15 models are developed with an increasing number (1 to 15) of model features included. Each of these models is developed 100 times, each time with a new data partitioning, and subsequently tested. The final determination of the performance of each model is calculated as the average of the 100 cycles.

It should be noted that the model compositions and the discriminatory powers achieved by the models depend slightly on the splits used to divide the data into training and independent test data. To eliminate these slight random effects associated with data partitioning as much as possible and to demonstrate the robustness of the approach used and its results, we performed data partitioning and subsequent model development a total of 100 times (each time with repartitioned training and independent test data). All performance values, without exception, were calculated as averages of these 100 cycles (i.e., using 100 different data splits for training and testing) based on the independent test data. We described the exact procedure in detail in Musigmann et al. [18]. In addition, we also summarized the whole process in a flow chart in Figure 2 for better understanding.

Since the described procedure with 100 repetitions can in principle lead to up to 100 slightly different models, we additionally created two final models that fixed only the 7 and 12 features, respectively, that were selected most frequently in the 100 runs previously conducted. Using these 7 (respectively 12) fixed features, the data partitioning, training of the models, and subsequent determination of performance with independent test data were also repeated 100 times to also be able to determine the robustness of the results obtained with these fixed models.

## 3. Results

### 3.1. Results of Comparing MRI Sequences to Predict ATRX Status

**Figure 3:**
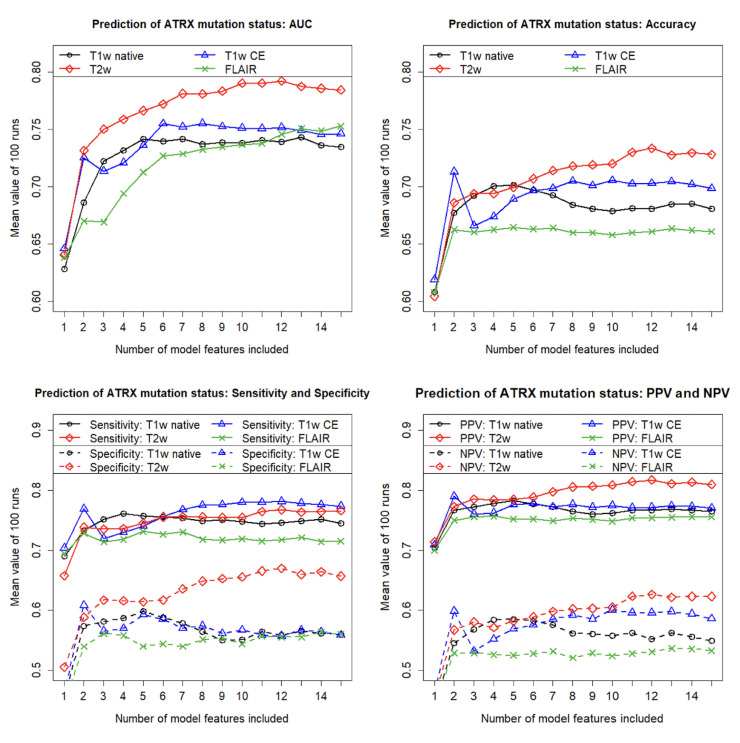
Predictability of ATRX mutation status for gliomas using different MRI sequences: Area under the curve (AUC), accuracy, sensitivity, specificity, positive predictive value (PPV), and negative predictive value (NPV) for the independent test samples, calculated as means of 100 repetitions (100 cycles) depending on the number of model features included. Feature pre-selection and model construction were performed using Lasso regression.

As explained earlier, all models were developed using Lasso regressions with an increasing number of features to predict the ATRX mutation status. To determine which of the commonly used MRI sequences is best suited for this task using radiomics-based models, we used the associated MRI images from each of these sequences to determine discriminatory power with respect to various metrics. Figure 3 shows the obtained discriminatory power in terms of AUC (top left), accuracy (top right), sensitivity and specificity (bottom left), and finally positive and negative predictive value (bottom right), depending on the number of features included in the models. All values were calculated with independent test data as averages of 100 runs, with each run involving a different data partitioning, a new feature selection, and a subsequent new model construction.

Regarding the four sequences compared, the FLAIR sequence is the least suitable for predicting ATRX mutations. When using the FLAIR sequence, the addition of further features already leads to almost no further increase in discriminatory power from the second feature onwards (except for the AUC).

Comparing the results obtained with the four sequences used, the discriminative powers obtained with the T1w sequences are in the middle range. Here, on average, administration of the contrast agent results in a slight increase in discriminatory power compared with the case in which native MRI images were used.

However, it is evident that the best results for the prediction of ATRX mutations were obtained with the T2w sequence. The T2w sequence almost always achieves the highest discriminatory power with respect to the different metrics studied. The only exception is sensitivity. Here, the discriminatory power achieved with the T1w CE sequence is minimally higher compared to the discriminatory power achieved with the T2w sequence. Overall, the best results are obtained in the case where the T2w sequence is used, and the models contain 12 features. For the models with even higher complexity, the discriminatory power decreases again, marking the beginning of overfitting when more than 12 features are included.

### 3.2. Results for the Final Models: Fixed Feature Model Approach

Since the models are completely newly developed each time for each of the total of 100 runs, they can differ in principle in their feature composition. Therefore, for each feature included in at least one of the 100 models, we determined the number of 100 runs in which the feature was selected. Table 2. lists, for each of the most frequently selected features, the number of 100 runs in which the feature was included in the 12-feature models (with respect to the T2w sequence). However, analysis of the features selected in the 100 runs indicates that the models are very similar in important parts of their feature composition. In this way, it can be seen that the four features “original shape Flatness”, “age at diagnosis”, “original first order Skewness” and “original gldm Dependence Variance” are included in all or almost all models. The two further features “original shape Least Axis Length” and “original glszm Small Area Emphasis” are included in more than 90 percent of all models, the feature “original glszm Gray Level Non Uniformity” in 82 percent of the runs, and the feature “original glszm Size Zone Non Uniformity Normalized” still in 79 percent of all runs.

Beyond these eight most frequently selected features, the importance of the other possible additional features quickly decreases sharply. This fact is consistent with the observation that for the T2w sequence, further additional features no longer significantly increase discriminatory power compared to a model with 7 or 8 features (see Figure 3).

To determine the extent to which the performance obtained with the models depends on the exact individual model composition, we additionally developed two models that included the features most frequently selected during the 100 runs in a fixed manner. These two models were based on the MRI images of the T2w sequence and included the most important 7 and 12 features, respectively. Thus, the model with seven included features contained only those features that were selected in at least 80 percent of the previously conducted runs (i.e., features “original shape Flatness” to “original glszm Gray Level Non Uniformity”, see Table 2). The model with the 12 most important features, on the other hand, contained exactly the number of features with which the highest performance had previously been achieved. Each of these two models was again developed 100 times with different data partitions. However, as described, this time the features included in the models were fixed during the 100 runs. Table 3 summarizes the classification results of our 7- and 12-feature models for predicting ATRX mutation status for the case where MRI images were acquired using the T2w sequence.

The “Different features” columns list the results for the models for which the features were redetermined in each of the 100 runs (according to the method used to calculate the results shown in Figure 3). Accordingly, the columns labeled “Fixed features” list the results for the models that were determined with the fixed features as shown in Table 2 All results were again calculated with independent test data and as mean values of 100 runs. The table also contains the 95 percent confidence intervals of the results. It is obvious that the additional features of the 12-feature model do not contribute significantly to an additional improvement in discriminatory power compared to the 7-feature model (compare Figure 3). The 7-feature models exhibit the following average performance (different features/fixed features): mean AUC = 78.1/83.1 percent, mean accuracy = 71.4/74.6 percent, mean sensitivity = 75.6/77.2 percent, mean specificity = 63.6/69.7 percent, mean PPV = 79.8/83.2 percent, and mean NPV = 59.8/63.6 percent. Thus, our models show high performance in predicting ATRX mutation status.

The described methodology with 100 repetitions is well-suited to investigate the stability of a particular model approach and to compare different approaches. In our case, the approach we chose yielded very stable results. The models developed with the different features had very similar discriminatory power compared to the models subsequently developed with fixed features. Our results show that our methodology for predicting ATRX mutation status using Lasso regression based on MRI images obtained with the T2w sequence leads to valid and stable results. The stability is reflected both in the performance achieved and in the respective model composition with respect to the features included. Our results depend only to a very small extent on random effects such as the data partitioning used. The ATRX status can be predicted with high accuracy by integrating the previously identified features into a single final model.

It should be noted here that it is, of course, conceivable in principle that using a machine learning algorithm other than Lasso regression, a different MRI sequence could prove to be best suited for predicting ATRX mutations. Therefore, we calculated the univariate discriminatory power of the respective 107 radiomic features for each of the four MRI sequences used. These values are independent of the machine learning algorithm that is subsequently used for model development. On average, the relevant features of the T2w sequence also showed the highest discriminatory power in these univariate analyses. Of course, this need not be the case simultaneously in a multivariate approach, but it can be interpreted as an additional indication that the T2w sequence is likely to provide the best results with respect to our diagnostic question.

In addition, we also developed models with a Lasso regression where we provided the features of all four sequences simultaneously for feature selection. The features that were selected first here were also part of the T2w sequence. Some of the subsequently added features belonged to other sequences but did not lead to a further significant increase in the achieved discriminatory power.

Finally, we also tested other machine learning algorithms such as Naive Bayes and linear discriminant analysis (LDA). These algorithms yielded slightly lower discriminatory power than Lasso regression. In the case of Naive Bayes, the best model resulted from having 13 features included. This model produced the following mean performance results: AUC = 0.733 (0.519, 0.912), accuracy = 0.712 (0.526, 0.900), sensitivity = 0.772 (0.538, 0.960), specificity = 0.601 (0.218, 0.925), PPV = 0.788 (0.630, 0.960), and NPV = 0.602 (0.311, 0.925). The best model with LDA was obtained with 9 features and yielded the following mean performance results: AUC = 0.730 (0.516, 0.901), accuracy = 0.691 (0.476, 0.850), sensitivity = 0.735 (0.462, 0.960), specificity = 0.607 (0.286, 0.857), PPV = 0.781 (0.619, 0.909), and NPV = 0.571 (0.318, 0.912). However, among these and the other machine learning algorithms we tested that also exhibited significant discriminatory power, the T2w sequence again proved to be the best overall for predicting ATRX mutations.

## 4. Literature Review

Recent studies showed that machine learning techniques enable reliable biomarker detection in glioma using imaging diagnostics. This was exposed by Chang et al. (2017), who demonstrated the utility of deep machine learning techniques applied to MRI to predict IDH mutation status in glioma grade II to IV (WHO Classification 2016) in a large multicentric study, achieving 82.8–85.7 percent accuracy [19].

In a similar manner, Zhou et al. (2019) analyzed preoperative MR images of over 500 glioma patients from three institutions, focusing on the sequences T1 CE and FLAIR, proving the ability to predict 1p19q codeletion status using machine learning algorithms with 68.5–71.6 percent AUC in low and high-grade gliomas [20].

Knowing that the IDH- and 1p19q codeletion status are not the only molecular characteristics of relevance or with an impact on the prognosis and therapy of glioma, Yan Ren et al. studied 2019 the prediction of ATRX expression loss in low-grade glioma using T2/FLAIR, diffusion-weighted imaging (DWI) derived apparent diffusion coefficient (ADC) and exponential ADC, among other MRI sequences, achieving an accuracy up to 91.67 percent [21].

The prediction of ATRX mutation status in glioma was also proved by Leng et al. [22] 2022 using a Lasso regression algorithm model and fifteen radiomics features on different MRI sequences, reaching an AUC on the training set of 0.93 and 0.84 on the validation set.

## 5. Discussion

After the literature review, we know that it is possible to predict the molecular status of different tumors using radiomics machine learning models in MRI. We decided to go one step further and determine how is the performance of each one of the normally indicated MRI sequences in predicting the ATRX mutation status in adult-type diffuse glioma, for this is such a relevant molecular marker determining the prognosis and possible line of treatment. Although it is obvious that molecular diagnosis can be reached in a biopsy, we are aware that in some clinical settings, it is not always possible to conduct a biopsy because of the previously mentioned risks of mortality, seizures, impaired consciousness, or hemorrhage. Likewise, it is not always possible to perform an MRI exam with all the recommended sequences (T1native, T1 CE, T2, FLAIR).

In this study, the cohort was divided into training and test datasets, and the most important radiomic features in each MRI sequence were selected using the VarImp-function. We developed a radiomics-based machine learning model using the Lasso regression algorithm and including the most important 7 and 12 features, which demonstrated that the best MRI sequence to predict ATRX mutation status in adult-type glioma is T2w, showing high performance and a mean AUC of 78.1 to 83.1 percent, a mean accuracy of 71.4 to 74.6 percent, a mean sensitivity of 75.6 to 77.2 percent, and a mean specificity of 63.6 to 69.7 percent.

We then proved the stability of this new model approach by calculating all values with independent test data as averages of 100 runs, in which T2w always achieved the highest discriminatory power. The results were also confirmed using other machine learning algorithms such as Naive Bayes and linear discriminant analysis, in which the T2w sequence again proved to be the best overall for predicting ATRX mutations.

Our study also discovered that the least suitable MRI sequence for this purpose is FLAIR. The T1w native sequence is in the middle range, reaching an improved performance in the contrast-enhanced sequences.

As previously mentioned, histological and molecular characteristics of adult-type diffuse gliomas are crucial for classification, prognosis, and treatment. According to the guidelines of the German Association of Neurology and Neurosurgery, the current recommendation for the treatment of gliomas depending on the WHO classification includes a multimodal concept with surgical removal or tumor reduction, chemotherapy, mostly with Temozolomid or Procarbazin, CCNU (Lomustine) and Vincristin, among others, and radiotherapy [23].

However, it was proven that the loss of ATRX expression inhibits cell growth and invasion, increasing the sensitivity of glioma cells to Temozolomide (TMZ), so a higher expression of ATRX leads to TMZ resistance [24]. These kinds of discoveries have led to a growing interest in the last few years regarding ATRX mutation status and to investigate possible new treatment approaches [25].

For instance, Pladevall-Morera et al. published in 2022 a screening of approved drugs by the Food and Drugs Administration (FDA) that are toxic to ATRX deficient cells and postulated that a combination of the current first-line chemotherapeutic agent TMZ and receptor tyrosine kinase inhibitor (RTKi) increases toxicity in ATRX deficient high-grade glioma cells, possibly improving the clinical outcome [26]. This demonstrates once again the relevance of being able to predict the ATRX mutation status and the status of other molecular markers in adult-type diffuse glioma, using common imaging technics and machine learning, which should become a part of the daily basis diagnosis.

To the best of our knowledge, this is the first time studying and comparing the individual performance of the usually conducted MRI sequences in the radiomics-based diagnosis of ATRX mutation status glioma. This is of great significance to the medical field, then using only one specific MRI sequence to make this molecular diagnosis may be of help in future years to economize classifying gliomas and simplify the therapeutical decisions based on molecular profile.

There are limitations that need to be addressed in this study. First, some information regarding the biomolecular status of the initially compiled patients was missing because of the retrospective character of the study. This led to excluding a relatively large number of patients. Second, studies with larger cohorts in multiple centers are required to validate our results.

## 6. Conclusions

In this study, we compared the performance of the different, commonly used MRI sequences using radiomics-based machine learning to predict the ATRX mutation status in adult-type diffuse glioma. Using the Lasso regression algorithm, we proved that the best-suited sequence is T2w, achieving an accuracy of up to 75 percent. These findings are significant in the medical field for simplifying the classification of adult-type diffuse glioma and choosing the right therapeutic path, as science explores new treatment approaches for this and other tumors based on their molecular characteristics.

## Figures and Tables

**Table 1:** Demographic characteristics of patients used to compare MRI sequences in terms of predictability of ATRX mutation status. The following sequences were used for all cases: T1w native, T1w CE (contrast-enhanced), T2w and Flair.

	Training Data	Independent Test Data	Total Data
Number of Patients	81	20	101
Gender in percentage			
Male	53.44	53.55	53.47
Female	46.56	46.45	46.53
Mean Age in years	42.98	43.25	43.04
ATRX Status in percentage			
Retained, not mutated	62.96	65.00	63.37
Lost, mutated	37.04	35.00	36.63

**Table 2:** Most important features for the T2 sequence included in the 12-feature models and their frequency of selection during 100 runs.

Level of Importance	Feature Name	Number of Runs Included
1	original shape Flatness	100
2	Age at dignose	99
3	original firstorder Skewness	98
4	original gldm DependenceVariance	97
5	original shape LeastAxisLength	92
6	original glszm SmallAreaEmphasis	91
7	original glszm GrayLevelNonUniformity	82
8	original glszm SizeZoneNonUniformityNormalized	79
9	original gldm DependenceNonUniformityNormalized	67
10	original shape SurfaceVolumeRatio	59
11	original gldm LargeDependenceHighGrayLevelEmphasis	47
12	original firstorder Kurtosis	36
13	original glszm GrayLevelNonUniformityNormalized	27
14	original shape Elongation	25
15	original glrlm ShortRunLowGrayLevelEmphasis	24

**Table 3:** Classification results for the prediction of ATRX mutation status for the 7- and 12-feature models. The columns labeled “Different features” show the results for the case that the features were newly determined in each of the 100 runs (i.e., the features included may be different). The results in the columns labeled “Fixed features” were calculated with fixed features according to Table 2. All results are calculated using independent test data and as mean values of 100 runs. The values in brackets indicate the 95 percent confidence interval.

	Models with 7 Features	Models with 7 Features	Models with 12 Features	Models with 12 Features
	Different Features	Fixed Features	Different Features	Fixed Features
AUC	0.781 [0.546:0.949]	0.831 [0.637:0.966]	0.792 [0.556:0.945]	0.829 [0.611:0.961]
Accuracy	0.714 [0.453:0.874]	0.746 [0.550:0.900]	0.734 [0.503:0.874]	0.752 [0.576:0.950]
Sensitivity	0.756 [0.425:0.923]	0.772 [0.502:0.923]	0.768 [0.538:0.960]	0.778 [0.538:1.000]
Specificity	0.636 [0.286:0.925]	0.697 [0.286:1.000]	0.670 [0.361:1.000]	0.703 [0.429:1.000]
PPV	0.798 [0.582:0.960]	0.832 [0.655:1.000]	0.817 [0.641:1.000]	0.835 [0.704:1.000]
NPV	0.598 [0.256:0.857]	0.636 [0.349:0.857]	0.627 [0.329:0.925]	0.652 [0.423:1.000]

## Data Availability

Data available on request. The data presented in this study are available on request from the corresponding author.

## Abbreviations

The following abbreviations are used in this manuscript:

CNS	Central Nervous System
WHO	World Health Organisation
MRI	Magnetic Resonance Imaging
FAIR	Fluid attenuated inversion recovery
ATRX	alpha thalassemia retardation syndrome X-linked
TMZ	Temozolomid
